# Hysteroscopic metroplasty for the treatment of the dysmorphic uterus: A SWOT analysis

**DOI:** 10.3389/fsurg.2022.1097248

**Published:** 2023-01-26

**Authors:** Maria Carrera, Luis Alonso, Jose Antonio Domínguez, Juan Luis Alcázar, Jose Carugno, Enrique Moratalla, Federico Pérez Milán, Miguel Caballero

**Affiliations:** ^1^ Hospital Universitario Doce de Octubre, Madrid, Spain; ^2^ Special Interest Group on Benign Reproductive Pathology of the Spanish Fertility Society, Spain; ^3^ Unidad de Cirugia Reproductiva, Centro Gutenberg, Málaga, Spain; ^4^ IERA, Badajoz, Spain; ^5^ Clínica Universidad de Navarra, Pamplona, Spain; ^6^ Obstetrics, Gynecology and Reproductive Sciences Department, Minimally Invasive Gynecology Division, University of Miami, Miller School of Medicine, Miami, FL, United States; ^7^ Hospital Universitario Ramón y Cajal, Madrid, Spain; ^8^ Hospital General Universitario Gregorio Marañón, Madrid, Spain

**Keywords:** dysmorphic uterus, hysteroscopic metroplasty, t-shaped uterus, infertility dysmorphic uterus, infertility, miscarriage, recurrent pregnancy loss, SWOT analysis

## Abstract

**Introduction:**

Dysmorphic uterus or T-shaped uterus is an increasingly frequent diagnosis among the infertile population that has been associated to worse reproductive results. Hysteroscopic metroplasty is a safe and simple procedure that can improve the reproductive outcomes in this group of patients, although the benefits of this procedure remains controversial due to the lack of adequate scientific evidence.

**Objective:**

To analyze the hysteroscopic metroplasty using the SWOT (Strengths, Weaknesses, Opportunities and Threats) methodology.

**Data sources:**

An electronic search from inception each database up to December 2021 including the following databases was conducted: PubMed-MEDLINE, EMBASE, Web of Science, The Cochrane Library, and Google Scholar.

**Methods of study selection:**

Studies reporting outcomes of patients undergoing hysteroscopic metroplasty were included.

**Tabulation:**

Not applicable

**Integration and Results:**

Clinical evidence from the included studies suggests an improvement in reproductive results after performing hysteroscopic metroplasty especially in women with recurrent pregnancy loss and previous infertility, but all of them have relevant methodological limitations. For this reason, benefits, risks and alternatives of this intervention should be considered with caution.

**Conclusions:**

Evidence from published data shows a probable association between dysmorphic uterus and poor reproductive outcomes. Hysteroscopic metroplasty in patients with dysmorphic uterus could improve pregnancy outcomes, but there is need of properly designed prospective controlled studies to determine the benefits of this technique.

## Introduction

Dysmorphic uterus, corresponding to the U1a class of the European Society of Human Reproduction and Embryology/European Society of Gynecologic Endoscopy (ESHRE/ESGE) classification (DU U1a), is an uncommon uterine malformation that was first described in 1977 in women exposed *in utero* to diethylstilbestrol (DES) by Kaufman et al. (1) and later by Buttram and Gibbons in 1979 (2). Since the Food and Drug Administration (FDA) banned the use of DES in 1971 (3), DU U1a has also been diagnosed in patients not exposed to DES, most frequently in patients with infertility. DU U1a is a Müllerian anomaly that can be congenital or acquired. When congenital, it is considered to be caused by lack of later development of the uterus (4). Although its origin is controversial, acquired forms have been associated with adenomyosis, advanced maternal age and the presence of intrauterine adhesions (5–8). DU U1a uterine malformation is characterized by a uterus with normal external contour with thickened lateral walls giving the characteristic appearance to the uterine cavity of T–shaped. This myometrium excess gives rise to a subcornual constriction ring which causes the hypoplasia of the uterine cavity (9–11).

The prevalence of dysmorphic uterus in the general population has not been determined (12) because women with dysmorphic uterus are usually asymptomatic. This anomaly is frequently diagnosed in women who consult for infertility, so its prevalence in the general population remains unknown. Also, the recognition of this rare condition has become more evident with the incorporation of 3D ultrasound imaging which allows a thorough visualization of the uterine cavity in the coronal view, making more evident its characteristics. In a systematic review recently published by Coelho-Neto et al. (13), the prevalence of dysmorphic uterus ranged between 0.2 to 10%, depending on the population studied.

Some controversial issues regarding dysmorphic uterus among the existing classification systems have generated conflict in its definition, resulting in added difficulty in establishing the prevalence of this malformation. T-shaped uterus was first included in 1988 American Association of Reproductive Medicine (ASRM) classification of uterine anomalies in class VII (DES-related) (14). Later, in 2013, the ESHRE-ESGE classification of Müllerian anomalies (15) included the T-shaped uterus in the class U1 or dysmorphic uterus. The new 2021 ASRM Müllerian anomalies classification (16) removed the class VII anomalies, as DES exposure is no longer occurring. However, having removed the entire class VII from the classification system excludes the dysmorphic uterus as a Müllerian anomalies.

Several studies (17, 18) have shown poor reproductive performance in patients with dysmorphic uterus. The pathophysiology explaining the poor obstetrical outcomes of patients diagnosed with dysmorphic uterus is not clear, although it might be related to a modified endometrial lining which would be responsible for lower implantation rates (12). Recently, an increasing number of studies evaluating the effect of hysteroscopic metroplasty in patients with dysmorphic uterus Alonso et al. (19), Adriaensen et al. (20), Boza et al. (6), Di Spiezio et al. (21), Ferro et al. (22), Giacomucci et al. (23), Haydardedeoglu et al. (24), Mounir et al. (25), Sanchez-Santiuste et al. (11), Sukur et al. (7) and Uyar et al. (26), have described an improvement of the reproductive outcomes after restoring the normal anatomy of the uterine cavity.

However, it is important to highlight that the evidence regarding the efficacy of this procedure must be considered of very low quality as the available studies are mainly retrospective, observational and lack a control group, as it has been highlighted in three recently published review papers by Garzon et al. (27), de Francinis et al. (28), and Coehlo-Neto et al. (13).

**Figure 1:**
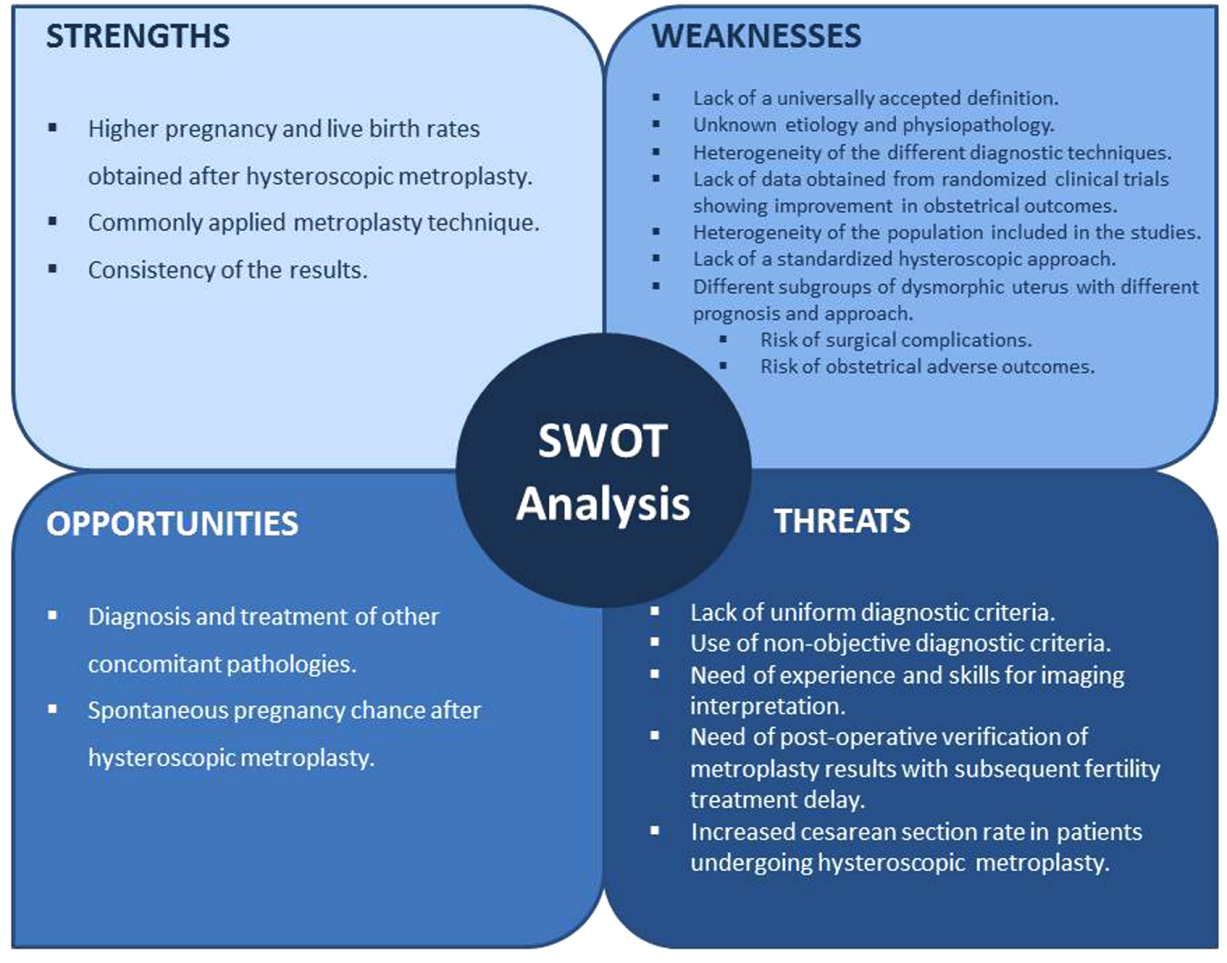
SWOT analysis of hysteroscopic metroplasty for the treatment of the dysmorphic uterus.

For the above mentioned reasons, we have conducted a SWOT (Strengths, Weaknesses, Opportunities and Threats) analysis to evaluate the available scientific evidence on the impact of hysteroscopic metroplasty in women not exposed to DES who are diagnosed with dysmorphic uterus and desire of future fertility (Figure 1).

## Strenghts

### Higher pregnancy rate and live birth rate obtained after hysteroscopic metroplasty

Several studies have assessed pregnancy and live birth outcomes in patients diagnosed of DU with previous poor reproductive outcomes as recurrent pregnancy loss, recurrent implantation failure and primary infertility. In order to evaluate all the available evidence on obstetrical outcomes after hysteroscopic metroplasty in women with dysmorphic uterus not exposed to DES we have performed a comprehensive review of the literature searching popular electronic databases (PubMed-Medline, Embase, Web of Science and Google Scholar) and performed a pooled analysis of the obstetrical outcomes. After the search, eleven studies were selected Alonso et al. (19), Adriaensen et al. (20), Boza et al. (6), Di Spiezio et al. (21), Ferro et al. (22), Giacomucci et al. (23), Haydardedeoglu et al. (24), Mounir et al. (25), Sanchez-Santiuste et al. (11), Sukur et al. (7) and Uyar et al. (26) for quantitative analysis. Table 1 show the main characteristics of the included studies. Pooled prevalence with 95% confidence intervals were obtained using MetaXL software (MetaXL, 5.3 https://www.epigear.com/index_files/metaxl.html epigear.com.) The global pooled pregnancy rate after hysteroscopic metroplasty was 68.9% (95% CI, 60.6% to 76.6%; 1,215 patients; 11 studies) and global pooled live birth rate was 56.2% (95% CI, 47.1% to 65%; 1,137 patients; 10 studies). In patients with history of recurrent pregnancy loss, pooled pregnancy rate after metroplasty was 78.9% (95% CI, 71.2% to 85.8%; 115 patients; four studies) and live birth rate was 62.8% (95% CI, 53.2% to 72%; 115 patients; four studies). In patients with primary infertility, the pooled pregnancy rate was 65.6% (95% CI, 56.7% to 73.9%; 790 patients; 8 studies) and live birth rate was 54.5% (95% CI, 44.8% to 64%; 484 patients; 6 studies). In patients with recurrent implantation failure the pooled pregnancy rate was 71.5% (95% CI, 56.2% to 90.3%; 34 patients; 2 studies) and live birth rate was 63.2% (95% CI, 22.3% to 96.3%; 34 patients; 2 studies. Forest plots are provided in Supplementary Figures S1–S8.

**Table 1:** Characteristics of included studies.

Reference	Country	Years	Study design	Diagnostic method	Diagnosis/classification	Total patients	In utero DES exposure	Primary infertility	Recurrent Pregnancy Loss	Implantation Failure
Alonso 2019	Spain	2015–2017	Prospective	3D TVUS and hysteroscopy	T-shaped uterus	63	None	27	20	16
Adriansen 2016	Belgium	–	Retrospective	–	Dysmorphic uterus class U1/ ESHRE-ESGE	103	None	85	18	–
Boza 2018	Turkey	2015–2017	Prospective	HSG and 3D TVUS	T-shaped uterus	56	None	32	10	14
Di Spiezio 2019	Multicentric	2011–2017	Retrospective	3D TVUS and hysteroscopy	class U1/ ESHRE-ESGE	214	None	166	48	0
Ferro 2018	Spain	2008–2015	Retrospective	Hysteroscopy	Dysmorphic uterus	190	None	0	0	190
Giacomucci 2011	Italy	2000–2006	Retrospective	Hysteroscopy	T-shaped uterus	17	–	0	0	17
Haydardedeoglu 2018	Turkey	2013–2016	Retrospective	HSG	Dysmorphic uterus class U1/ ESHRE-ESGE	272	None	162 (Secondary infertility:110)		
Mounir 2012	Egypt	–	Retrospective	HSG/3D TVUS	T shaped uterus	88	–	38		50
Sanchez-Santiuste 2020	Spain	2016–2020	Retrospective	Hysteroscopy	Dysmorphic uterus	63	None	30	33	–
Sukur 2018	Turkey	2009–2015	Retrospective	HSG/MRI	Dysmorphic uterus class U1/ ESHRE-ESGE	78	None	43	35	–
Uyar 2019	Turkey	2011–2016	Retrospective	HSG/3D TVUS	T shaped uterus	101	None	101	–	–
Characteristics of included studies—Surgical procedure										

Reference	Metroplasty procedure	Anti-adhesion Agents	Hysteroscopic 2nd look/Other metohod	Time to 2nd look (months)	Time until pregnancy allowed (months)	Complications	Follow-up (months)			
Alonso 2019	5-mm hysteroscope and 5-Fr operative scissors	No/ sequential Estroprogestins	Yes	3	3	None	16 (12–44)			
Adriansen 2016	5-mm hysteroscope and 5-Fr operative scissors	–	Yes	2	–	None	–			
Boza 2018	5-mm hysteroscope and 5 Fr bipolar electrode	No/sequential Estroprogestins	No/HSG/3D TVUS	HSG (1) and 3D TVUS (1,2,6,12)	2	None	12			
Di Spiezio 2019	4- to 5-mm hysteroscopes and 5 Fr bipolar electrode or scissors	Yes	Yes	2	–	16.8% synechiae	60 (24–96)			
Ferro 2018	4.2-mm hysteroscope and 5 Fr bipolar electrode or scissors	No/ sequential Estroprogestins	Yes	1	1	None	12			
Giacomucci 2011	5-mm hysteroscope	–	–	–	–	–	–			
Haydardedeoglu 2018	5-mm hysteroscope and 5 Fr bipolar electrode	No/Estradiol Valerate	No/HSG	2	–	–	–			
Mounir 2012	Monopolar resectoscope	No/2 months	–	–	2	–	–			
Sanchez-Santiuste 2020	–	–	–	–	–	–	9–48			
Sukur 2018	Monopolar resectoscope	No/-	Yes	2	2	–	27 (12–84)			
Uyar 2019	Monopolar resectoscope	No// sequential Estroprogestins	–	–	–	–	–			

HSG, Hysterosalpingography; 3D TVUS, 3 dimensional transvaginal ultrasound.

The described results indicate a clear improvement in the obstetrical outcomes of patients with dysmorphic uterus after hysteroscopic metroplasty. All the included studies reported the live birth rate before and after the hysteroscopic metroplasty revealing a dramatic increase. The average live birth rate before the metroplasty was under 2%. After the procedure, the average live birth rate was over 55%, with some series showing live birth rates as high as 78% (22). Regarding the miscarriage rate, there was a drop in the prevalence from over 85% before the hysteroscopic metroplasty to 20% after the surgical procedure. In summary, it seems that the main strength of the available scientific evidence regarding the impact of hysteroscopic metroplasty in patients diagnosed with dysmorphic uterus is that performing hysteroscopic metroplasty is associated with increased live birth rate and decrease pregnancy loss rate.

### Uniform metroplasty technique

In all the included studies except one (21), the hysteroscopic metroplasty of dysmorphic uteri was performed using a similar technique. It consisted of incisions on the lateral uterine wall in order to obtain a triangular shaped uterine cavity, increasing the distance between lateral uterine walls at mid-cavity. This lateral wall incision was performed under direct vision and with a maximum incision depth of 7 mm and the surgery was completed when both tubal ostia were simultaneously visible from the isthmus. It is important to emphasize that despite that there is not a surgical technique accepted as the standard of care, the goal of the surgery in all cases was similar, aiming to enlarge the uterine cavity with the objective of getting a triangular shaped cavity (19).

### Consistency of the results and operator's experience

All the included studies were performed by experienced hysteroscopic surgeons in the field of reproductive surgery from different parts of the world, which supports the consistency of the results.

## Weaknesses

### Lack of a universally accepted definition

An important source of heterogeneity is the absence of a consensus regarding the definition of dysmorphic uterus. Among the studies included in our systematic review, seven used the ESHRE-ESGE classification Adriaensen et al. (20), Di Spiezio et al. (21), Ferro et al. (22), Giacomucci et al. (23), Haydardedeoglu et al. (24), Sanchez-Santiuste et al. (11) and Sukur et al. (7) while the remaining studies used the generic term of T-shaped uterus Alonso et al. (19), Boza et al. (6), Mounir et al. (25), and Uyar et al. (26) without a clear definition. Recently, Congenital Uterine Malformation by Experts (CUME) group have published 3D ultrasound criteria to define T–shaped uterus (29), but until now, none of the published series have used this classification.

### Unknown etiology and physiopathology

Another challenge is the fact that the etiology of the T-shaped uterus remains largely unknown. First reports of a T-shaped uterine malformation, which were made in women exposed *in utero* to dietilestilbestrol, date from 1977 (1). DES is a nonsteroidal synthetic estrogen that was used in the 1940s and 1950s aiming to prevent obstetrical complications in pregnant women. It caused several congenital malformations in the female fetus exposed to it *in utero* such as T-shaped uterus and Fallopian tube dysfunction, it is also associated with an increased risk of clear-cell adenocarcinoma of the vagina (30). Although DES was removed from the market in 1971 (13), the incidence of patients diagnosed with dysmorphic uterus is still increasing. Several studies (21, 31, 32) have considered a primary origin with a poorly understood etiopathology while others consider this anomaly to be secondary to intrauterine adhesion formation (6–8) or also to adenomyosis (5). The underlying biological mechanism causing worse reproductive outcomes in patients with congenital Müllerian anomalies remains uncertain. Among the hypotheses described, one that stands out is having an altered endometrial lining which would be responsible for lower implantation rates (12), moreover, an altered shape of the uterine cavity with a reduced volume might impair endometrial receptivity and diminish uterine growth (17, 18, 33).

### Heterogeneity of the different diagnostic modalities

Hysterosalpingography was the first imaging modality used to diagnose a T-shaped uterus. Currently, there are two preferred methods for diagnosing uterine anomalies, Magnetic Resonance Imaging (MRI) and 3D transvaginal ultrasound (3D-US), as both provide a coronal view of the uterine cavity. Due to the simplicity and accessibility of the 3D US technology, it is becoming the diagnostic modality most commonly used for the diagnosis of Müllerian anomalies.

In our review, five of the eleven included studies used 3D transvaginal ultrasound Alonso et al. (19), Boza et al. (6), Di Spiezio et al. (21), Mounir et al. (25) and Uyar et al. (26) alone or in combination with other diagnostic modalities as hysteroscopy Alonso et al. (19) and Di Spiezio et al.(21) or hysterosalpingography Boza et al. (6), Mounir et al. (25) and Uyar et al. (26). In three of them, hysteroscopy was the diagnostic modality of choice Ferro et al. (22), Giacomucci et al. (23) and Sanchez-Santiuste et al. (11), one study used hysterosalpingography as the diagnostic method Haydardedeoglu et al. (24) and another one combined hysterosalpingography with MRI ((7).

### Lack of data obtained from randomized clinical trials showing improvement in obstetrical outcomes

Despite the increasing number of publications Alonso et al. (19), Adriaensen et al. (20), Boza et al. (6), Di Spiezio et al. (21), Ferro et al. (22), Giacomucci et al. (23), Haydardedeoglu et al. (24), Mounir et al. (25), Sanchez-Santiuste et al. (11), Sukur et al. (7) and Uyar et al. (26) reporting improved obstetrical outcomes after hysteroscopic metroplasty, there is lack of solid scientific evidence regarding beneficial effects of this technique in patients with infertility or recurrent pregnancy loss, since there is no randomized controlled trial addressing this issue. All the available studies are observational, the vast majority are retrospective, excluding Alonso et al. (19) and Boza et al. (6), and lack of a control group.

### Heterogeneity of the population included in the studies

Another important limitation is that the published studies reporting obstetrical outcomes after hysteroscopic metroplasty included a heterogeneous group of women, some of them with diagnosis of primary infertility Alonso et al. (19), Adriaensen et al. (20), Boza et al. (6), Di Spiezio et al. (21), Haydardedeoglu et al. (24), Mounir et al. (25), Sanchez-Santiuste et al. (11), Sukur et al. (7) and Uyar et al. (26), others with recurrent pregnancy losses Alonso et al. (19), Adriaensen et al. (20), Boza et al. (6), Di Spiezio et al. (21),Sanchez-Santiuste et al. (11) and Sukur et al. (7) and some with implantation failure after IVF attempts Alonso et al. (19), Boza et al. (6), Ferro et al. (22), Giacomucci et al. (23), Mounir et al. (25). This makes it difficult to ascertain in which group or groups of patients metroplasty will be more beneficial.

### Lack of a standardized hysteroscopic approach

Another source of bias is that the metroplasty was performed using different hysteroscopic tools. Currently, the use of hysteroscopic 5 French scissors and normal saline as the distention media represents the most common technique Alonso et al. (19), Adriaensen et al. (20), Boza et al. (6), Di Spiezio et al. (21), Ferro et al. (22) and Haydardedeoglu et al. (24) although there are studies in which the metroplasty was performed with monopolar resectoscope Mounir et al. (25), Sukur et al. (7) and Uyar et al. (26). Despite the majority of the studies use a similar technique, there is not enough evidence to select a particular technique as the standard of care.

### Different subgroups of dysmorphic uterus with different prognosis and approach

Alonso et al. (34) have described different subtypes of dysmorphic uterus as T, Y or I shaped. It is unclear if all of them are suitable for the same metroplastic approach and if the postoperative results are similar in all cases.

### Risk of surgical complications

Another threat present in any surgical procedure is the risk of complications. In light of the data published in the literature we can consider that intraoperative complications during metroplasty for dysmorphic uterus are rare. Ducellier-Azzola et al. (31) in one of the most extensive series published (112 patients during 24 years) did not report any case of uterine perforation, although some cases have been reported (9). The most frequently reported complication as a result of hysteroscopic metroplasty is postoperative adhesion formation. In 2001, Aubriot et al. (35) reported a 33% rate of post procedure intrauterine adhesion formation in their series of 51 patients, although most likely, all these patients had in-utero DES exposure. Ducellier-Azzola et al. (31), in their series, described only a 2.7% postoperative adhesion formation rate (10).

With regard to safety, it is well known that expert high volume surgeons have better surgical outcomes and lower complication rates for any surgical procedure (36).

### Risk of obstetrical adverse outcomes

The incidence of obstetrical complications such as late pregnancy loss or premature delivery was not augmented by the hysteroscopic metroplasty. An important aspect to consider is whether pregnant patients who have had hysteroscopic metroplasty before conception would benefit from an elective cesarean section as a delivery route. Although vaginal delivery is not contraindicated, a high rate of cesarean sections (33%–61%) has been reported in patients with history of metroplasty (8, 21, 31, 35). This is probably due to the fear of uterine rupture in patients with history of uterine surgery. Other potential obstetrical complications are cervical insufficiency (31), abnormal placentation, post-partum hemorrhage (described in slightly more than 1% of patients) or peripartum infection in 2.6% (7).

## Opportunities

### Diagnosis and treatment of other concomitant pathologies

There has been a considerable debate on the role of office hysteroscopy in the work up of infertile patients before Assisted Reproductive Techniques (ART) (37). A recent systematic review with meta-analysis published by Mao et al., in 2019 [37], evaluating the effectiveness of performing hysteroscopy before ART in women with recurrent implantation failure, showed an increase in the chances of embryo implantation and pregnancy in patients undergoing hysteroscopy. Hysteroscopy allows to diagnose subtle lesions of the uterine cavity such as adhesions, polyps or fibroids that could be overlooked using transvaginal ultrasound and allows the treatment of those lesions in the same procedure (38, 39). It also provides a thorough visual inspection of the uterine cavity so the diagnosis of other pathologies as chronic endometritis can be ruled out. A recent SWOT analysis assessing the impact of chronic endometritis on fertility (40) concluded that in cases of recurrent pregnancy loss or repeated implantation failure the investigation and treatment of this entity could improve ART results.

### Spontaneous pregnancy rate after hysteroscopic metroplasty

Several authors, as Di Spiezio et al. (21) and Alonso et al. (19) have described a high spontaneous pregnancy rate after metroplasty. Alonso et al. (19) prospectively recruited a group of nulliparous women without other infertility factors and demonstrated a significant improvement of spontaneous pregnancy rate. This improvement can be explained by the change in uterine morphology, an increase in cavity size and the enhancement of the vascularization, despite the ultimate mechanism is not well known.

## Threats

### Lack of uniform diagnostic criteria

One of the main threats is the lack of a consensus for the diagnosis of dysmorphic uterus. This terminology was established by the ESHRE-ESGE classification, corresponding to the Class U1 anomaly (15) including mainly to the T-shaped uterus. This type of anomaly was already described in the AFS classification as Class VII (DES drug related) (14), which was subsequently removed in the new ASRM (16) (former AFS) classification.

### Use of non-objective diagnostic criteria

The diagnosis proposed by ESHRE-ESGE and the former AFS classifications is based on subjective impression of the visual analysis of the coronal view of the uterus (either by MRI, 3D-US or even HSG). This generates a high risk of biased interpretation and high inter-observer variability when establishing the diagnosis. In an attempt to overcome this problem, some authors have proposed objective criteria for establishing the diagnosis of dysmorphic uterus (29). However, these criteria have not been validated, and have not been widely adopted in clinical practice.

### Need of experience and skills for imaging interpretation

Another threat is related to the expertise of those who obtain and interpret the images of patients with dysmorphic uterus. To date, there is no evidence about the learning curve for achieving competency for this specific diagnosis.

### Need of post-operative verification of metroplasty results with subsequent fertility treatment delay

Several studies in the literature have tried to establish the optimal interval between hysteroscopic surgery and embryo transfer, but there are no studies that address this issue after metroplasty for dysmorphic uterus. It is clear that the endometrial healing time will depend on the procedure performed. After polypectomy, it has been seen that intervals greater than 120 days do not provide advantages and are even associated with worse biochemical and clinical pregnancy rates (41). Another study compared transfer during the following cycle after hysteroscopic polypectomy with transfer after 2, 3 or more than 3 cycles, and the gestational results (implantation rate, biochemical pregnancy rate and abortion rate) in the 3 groups were similar (42). Some studies analyzed transfer results in the same cycle of polypectomy and found that the results were not affected if more than 5 days passed between hysteroscopy and transfer (43, 44). But this interval should probably be longer after surgeries such as septoplasty or adhesiolysis since the endometrial damage in these cases is more extensive and probably similar to that produced after metroplasty for dysmorphic uterus. Berkkanoglu et al. (45) found no differences in pregnancy rate, cumulative pregnancy rate, implantation rate or miscarriage rate, in patients who had transfers during the first 10 weeks after septoplasty compared to those who had transfer between 10 and 17 weeks or after 17 weeks after the procedure. Yang et al.(46) monitored the endometrial lining after different hysteroscopic surgeries and found that 86% of women achieved a fully healed endometrium 1 month after polypectomy, a higher rate than those after myomectomy (18%), septal incision (19%), and adhesiolysis (67%). Postoperative office hysteroscopy revealed that 88% and 76% of the women had new intrauterine adhesions formation after septal incision and adhesiolysis, respectively, more than those after myomectomy (40%) and polypectomy (0%). Deng et al. (47) established that after adhesiolysis due to intrauterine adhesions, the optimal waiting period before transfer should be between 90 and 180 days compared to less than 90 days. All these data support the idea that second-look hysteroscopy should be performed 1–2 months after metroplasty and in some cases adhesiolysis would be needed, which can lead to a significant delay in fertility treatment in these patients.

### Increased cesarean section rate in patients undergoing hysteroscopic metroplasty

In patients undergoing hysteroscopic metroplasty, attempting a normal vaginal delivery is not contraindicated, even if the uterus could be considered scarred. Unfortunately, very high rates for cesarean section in patients with a history of hysteroscopic metroplasty who subsequently conceived are reported in the literature (33% to 57%). We have also confirmed this finding with a striking 61% cesarean section rate (8, 21, 31, 35). These rates could be due to excessive caution executed by the medical team and requested by the patients, some of them who have already had bad obstetrical outcomes in previous pregnancies. The most common feared risk is uterine rupture. However, only a single case has ever been reported (48).

## Conclusions

Over the last decade, dysmorphic uterus has gained attention in the field of reproductive medicine, being increasingly referred as a potential cause of fertility impairment. From the perspective of the actual definition, this anomaly shows notorious differences with its precursor DES derived T-shaped uterus.

Lack of consensus of a universally accepted diagnostic criteria of this anomaly limits the accuracy of its clinical characterization, and perhaps the validity of analytical studies assessing the potential effects on reproductive outcomes.

Currently, there is no high-quality scientific evidence that supports performing hysteroscopic metroplasty in patients with DU U1a, although the case series included in this review suggest that in patients with previous adverse obstetric outcomes this minimally-invasive procedure can improve pregnancy and live birth rates.

Although the efficacy of this technique needs to be confirmed with properly conducted prospective randomized controlled studies, due to its simplicity and low complication rates, hysteroscopic metroplasty could be offered to patients with a DU U1a and history of poor reproductive outcomes, provided a thorough discussion with the patient.

## Author contributions

Responsible for the document drafting and revision. All authors contributed to the article and approved the submitted version.
