# Supplementary figures and images for: Hysteroscopic metroplasty for the treatment of the dysmorphic uterus: A SWOT analysis
Source: Front Surg. 2023 Jan 26;9:1097248. doi: 10.3389/fsurg.2022.1097248 (PMC9909195; doi:10.3389/fsurg.2022.1097248)

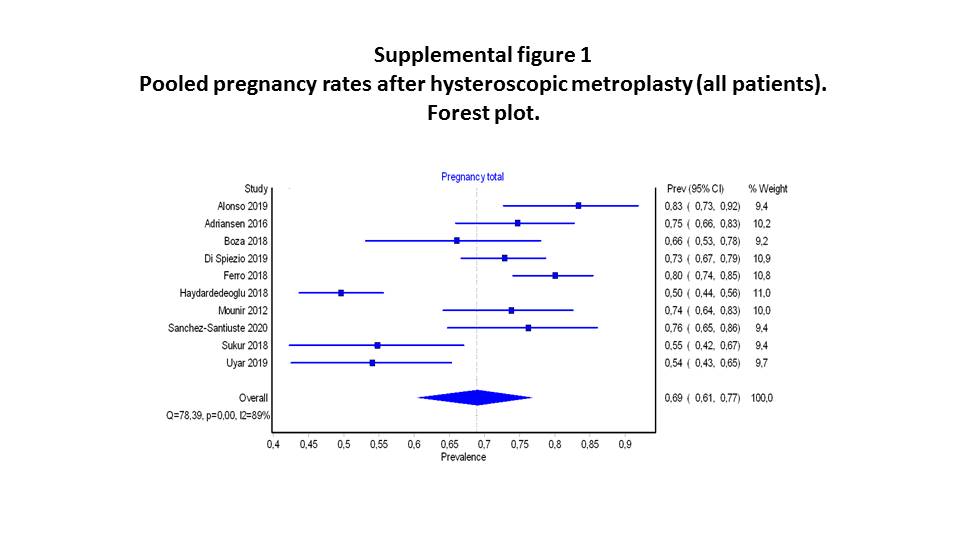

Supplement: Supplementary file 1 [file Image1.jpeg]

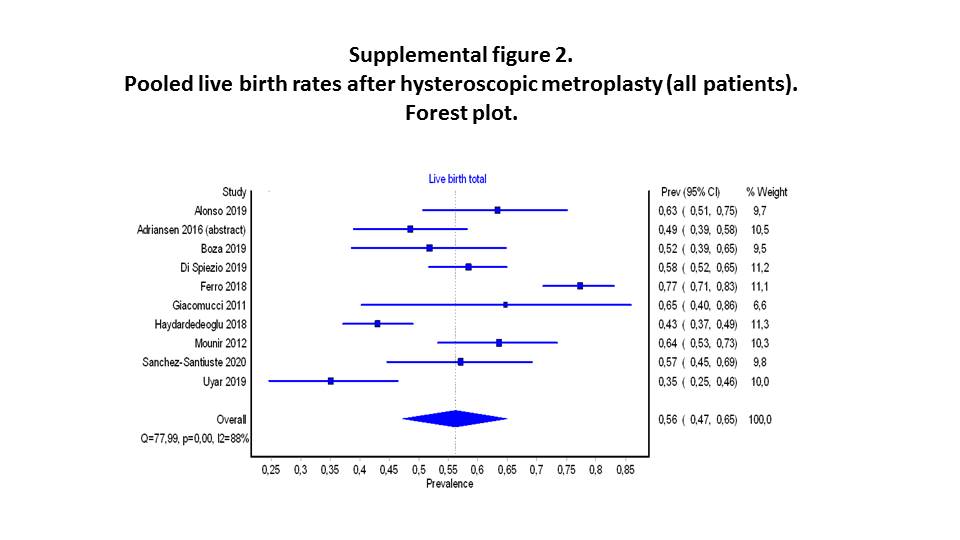

Supplement: Supplementary file 2 [file Image2.jpeg]

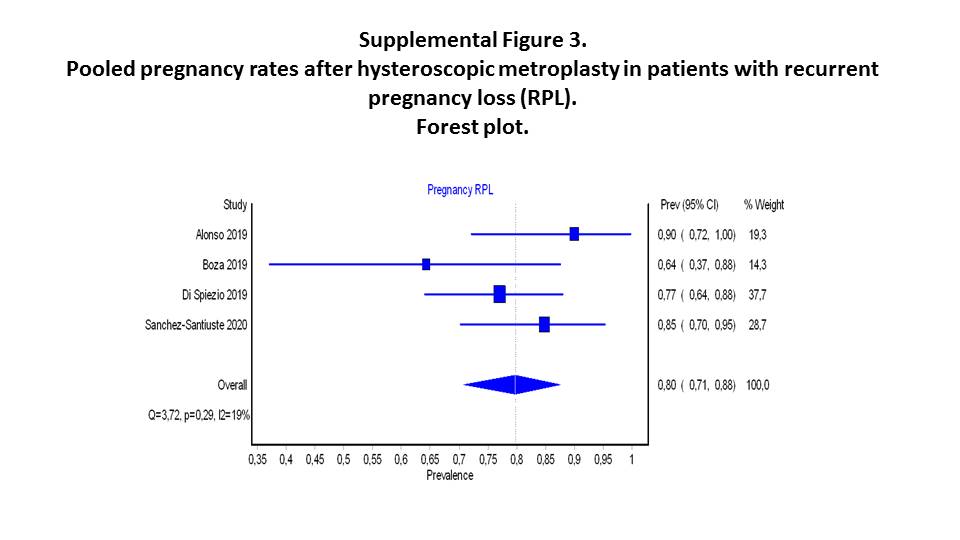

Supplement: Supplementary file 3 [file Image3.jpeg]

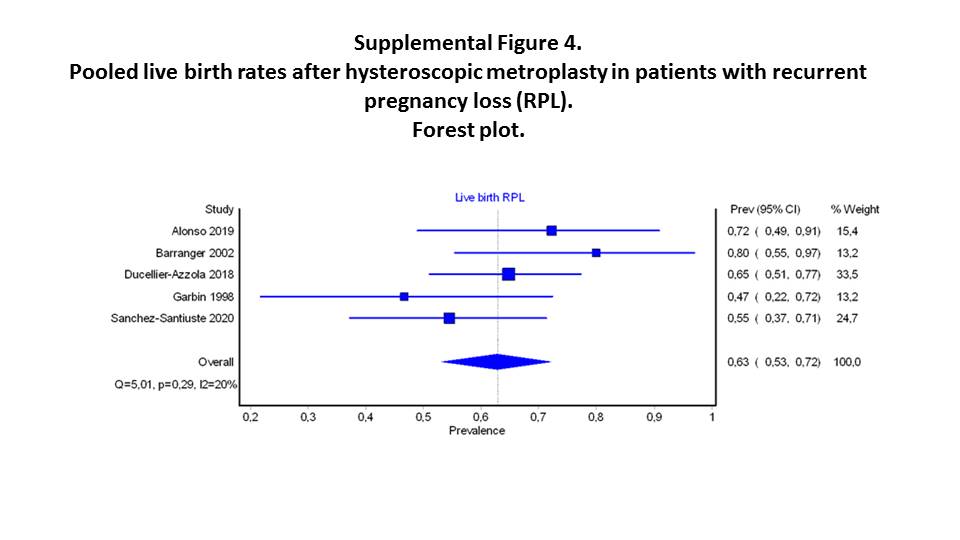

Supplement: Supplementary file 4 [file Image4.jpeg]

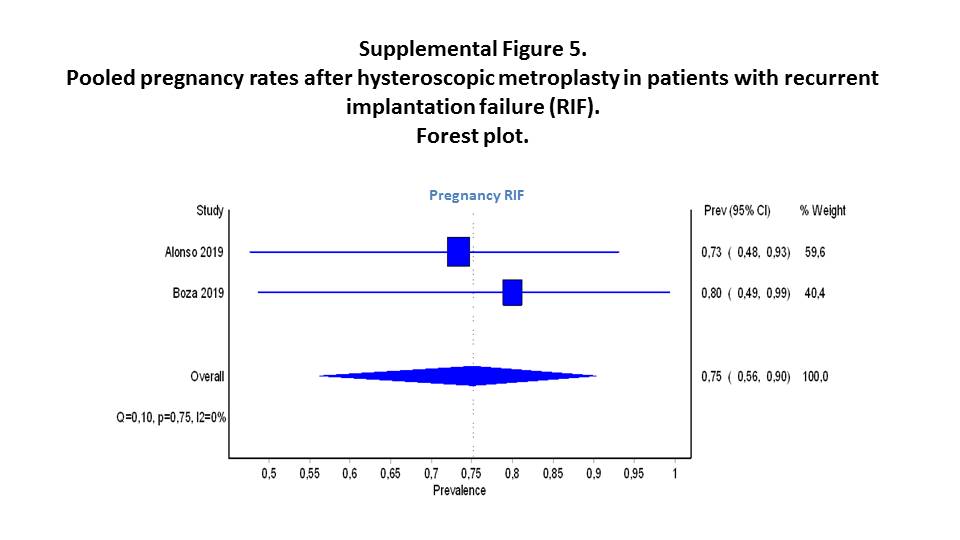

Supplement: Supplementary file 5 [file Image5.jpeg]

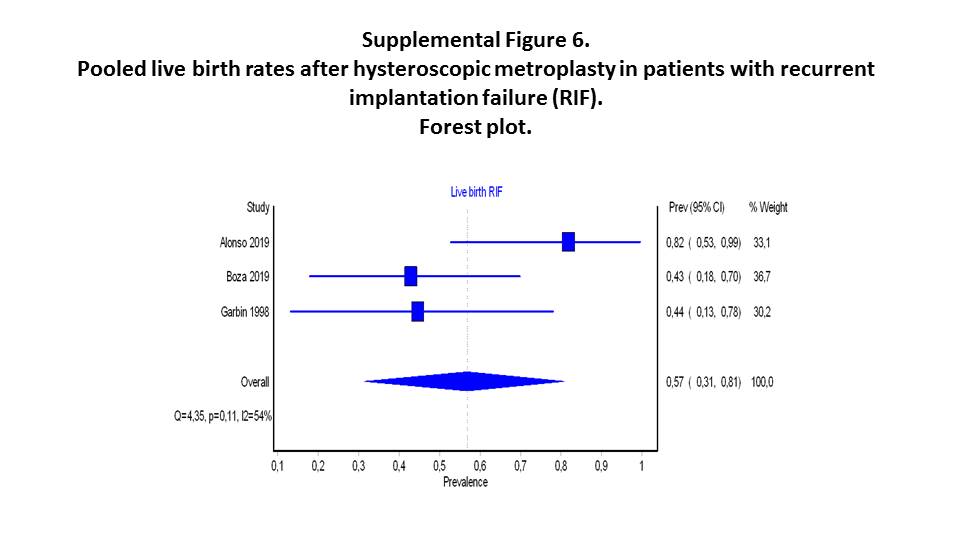

Supplement: Supplementary file 6 [file Image6.jpeg]

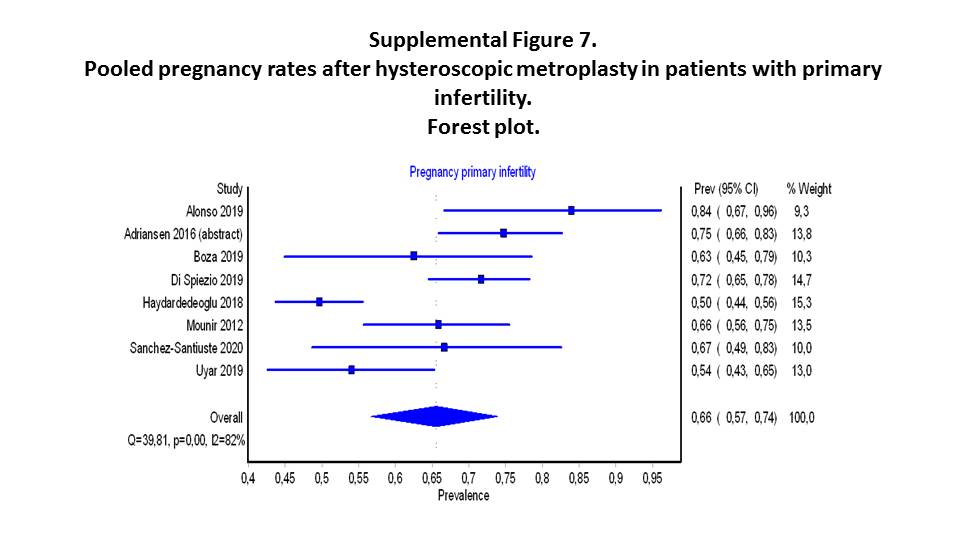

Supplement: Supplementary file 7 [file Image7.jpeg]

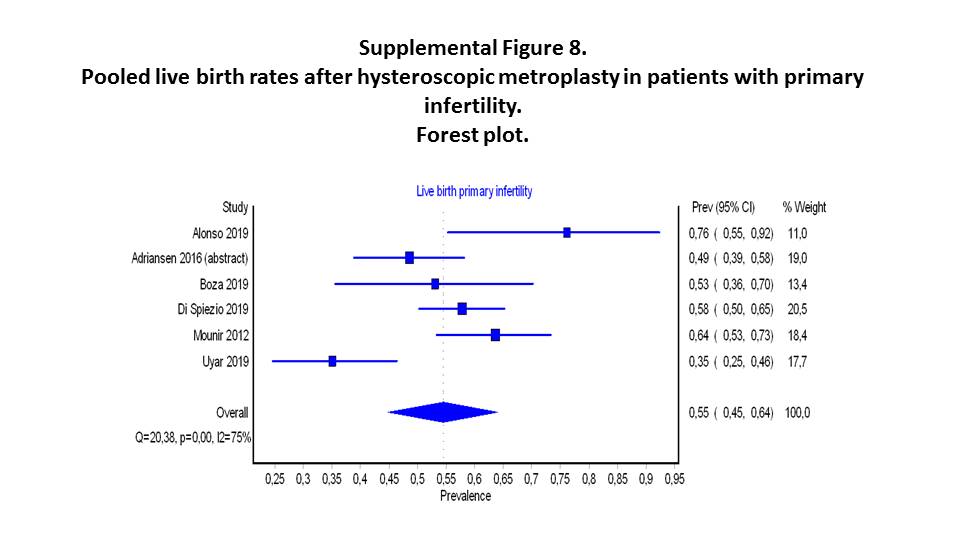

Supplement: Supplementary file 8 [file Image8.jpeg]
